# Exploring the Perspectives and Experiences of Older Adults With Asthma and Chronic Obstructive Pulmonary Disease Toward Mobile Health: Qualitative Study

**DOI:** 10.2196/45955

**Published:** 2023-08-22

**Authors:** Andrew Kouri, Samir Gupta, Sharon E Straus, Joanna E M Sale

**Affiliations:** 1 Women's College Hospital Toronto, ON Canada; 2 Unity Health Toronto St. Michael’s Hospital Toronto, ON Canada; 3 Institute of Health Policy, Management and Evaluation Dalla Lana School of Public Health University of Toronto Toronto, ON Canada; 4 Department of Surgery Faculty of Medicine University of Toronto Toronto, ON Canada

**Keywords:** older adults, mHealth, asthma, chronic obstructive pulmonary disease, qualitative research, digital health, qualitative study, airway disease, barrier, health technology, interview, smartphone, airway, implementation, mobile phone

## Abstract

**Background:**

The use of mobile health (mHealth) in asthma and chronic obstructive pulmonary disease (COPD) is growing, and as the population ages, a greater number of older adults stand to benefit from mHealth-enhanced airway disease care. Though older adults are a heterogeneous population of health technology users, older age represents a potential barrier to health technology adoption, and there is currently a lack of knowledge on how older age influences mHealth use in asthma and COPD.

**Objective:**

In this qualitative study, we sought to explore the experiences and perspectives of adults who were aged 65 years and older with asthma and COPD toward mHealth use.

**Methods:**

Semistructured individual interviews were conducted with adults who were aged 65 years and older with asthma or COPD and owned a smartphone. Applying phenomenological methodology, we analyzed interview transcripts in order to develop themes and propose an essential experience of mHealth use among older adults with airway disease. We then summarized our qualitative findings and proposed strategies to leverage our results in order to guide future research and implementation efforts targeting older adults’ use of airway mHealth.

**Results:**

Twenty participants (mean age 79.8, SD 4.4 years) were interviewed. Participants described a central tension between (1) the perception that mHealth could help maintain independence throughout aging and (2) an apprehension toward the ways in which mHealth could negatively affect established health care experiences. Several elements of these 2 themes are absent from previous research focusing on younger adults with asthma and COPD. The individual elements of these 2 themes informed potential strategies to optimize future older adults’ use of asthma and COPD mHealth tools.

**Conclusions:**

Focusing on the perspectives and experiences of older adults with asthma and COPD in their use of mHealth identified novel understandings of health technology use in this important demographic in need of greater care. These lessons were translated into potential strategies that will need to be objectively evaluated in future airway mHealth research, development, and implementation efforts.

## Introduction

Mobile technologies are increasingly being used in the care of people with asthma and chronic obstructive pulmonary disease (COPD), the 2 most common chronic airway diseases [1-4]. Broadly termed mobile health (mHealth), these may be smartphone or tablet apps, portable digital devices such as smart inhalers, home spirometers, and smart oximeters [1,5]. mHealth tools are particularly well suited for the needs of patients with asthma and COPD, which involve significant self-monitoring and self-management, regular use of inhaler devices, and frequent physiological reassessment [3,6]. Systematic reviews of the clinical effectiveness of mHealth in asthma and COPD have demonstrated improvements in quality of life, inhaler adherence, and asthma control levels, as well as reductions in emergency room visits [7-9]. The COVID-19 pandemic has also highlighted the promise of respiratory mHealth, as virtual care increased and in-person pulmonary function testing capacity was severely restricted [10].

As the global population ages, the proportions of patients with asthma and COPD above the age of 65 years have also risen [11,12]. Older adults with asthma and COPD represent a growing demographic that may greatly benefit from mHealth technology. For example, older adults have more difficulty with chronic respiratory disease self-management and lower levels of inhaled medication adherence, contributing to a higher risk of asthma- and COPD-related morbidity and mortality than their younger counterparts [13-16].

Though older adults are not a homogeneous population, and many older adults are interested and engaged with technology, older age may also be an important barrier to health technology adoption. For example, digital health literacy levels are generally lower among older adults, and older adults experiencing chronic medical conditions associated with physical and cognitive barriers present unique challenges to health technology use [17,18]. A number of studies have explored the possible barriers and facilitators to mHealth use among older adults with other chronic conditions [19-21], but none has specifically investigated the unique experiences of older adults with asthma and COPD.

The purpose of this qualitative study was to fill this knowledge gap by exploring the perspectives and experiences of adults who were 65 years and older with asthma or COPD in relation to their use of mHealth. This may help optimize future mHealth for this important and growing population of patients with chronic airway disease, and facilitate the development of future research elucidating the clinical benefits of mHealth specifically among older adults.

## Methods

### Qualitative Approach

As our goal was to better understand the experiences and perspectives of older adults, we chose a phenomenological methodology [22]. Phenomenology is a qualitative methodology that helps illuminate the essential elements of individuals’ experiences [23,24]. Phenomenology is focused on the individual and assumes that deeper meaning can be discerned through discovery-oriented analysis of detailed descriptions of lived experience [23]. It also explicitly acknowledges the role and influence of the researcher (see Multimedia Appendix 1 for further details). Our approach was guided by Giorgi’s [24-26] practical adaptation of Husserlian phenomenology for systematic scientific analysis, which is based around understanding the essence of a phenomenon by studying people engaging with it as part of their everyday lives. Giorgi’s [24,25] approach includes a systematic approach to data collection, analysis, and synthesis suited to scientific analysis, and emphasizes the importance of remaining engaged and reflective as an investigator. This qualitative study was reported in line with the COREQ (Consolidated Criteria for Reporting Qualitative Research) reporting guidelines where appropriate [27].

### Study Setting and Patient Recruitment

We recruited patients from a preexisting research database at a tertiary care respirology clinic at St. Michael’s Hospital, Unity Health Toronto, in Toronto, Canada (see Multimedia Appendix 1 for further details). Patients were included if they were English-speaking adults who were aged 65 years or older with a physician diagnosis of asthma or COPD (we also included patients if they reported having both conditions) and currently owned a smartphone. We used maximum variation sampling to target a range of ages above 65 years and a balance across sexes and between airway diagnoses. We planned for a sample size of 20 patients to increase the chance of qualitative data saturation, meaning that no new themes or analytic codes were developed from further data collection [28].

### Data Collection

We conducted individual, semistructured interviews to understand participants’ perspectives of mHealth. As this study took place during the COVID-19 pandemic, interviews were held virtually using Zoom (Zoom Video Communications) or telephone. AK conducted interviews between March 2021 and March 2022. Interviews lasted 40-60 minutes and were transcribed verbatim from audio recordings. An interview guide was created by AK and revised with coauthors. It included broad, open-ended questions around participants’ previous experiences with mHealth, how age influenced their perspectives of mHealth, and their general experiences with mobile technology (see Multimedia Appendix 1). Transcripts were uploaded in NVivo 12 (QSR International), qualitative data management software, to facilitate data analysis. A demographic questionnaire was also administered after each interview.

### Data Analysis

Data analysis occurred concurrently with data collection. AK coded and analyzed the data independently, as is customary in phenomenological studies, but also discussed results with JEMS (an experienced qualitative researcher) and coauthors throughout the study. Data analysis followed the steps outlined by Giorgi [24]. Transcripts were first read in full in order to achieve immersion in participants’ experiences. Transcripts were then reread and statements that conveyed the meanings of participants’ lived experiences were documented as meaning units (codes). Following this, codes relevant to the research objective were grouped together into themes. These themes were then reviewed, exploring the relationships between them, considering negative cases, and viewing them from the perspective of the research question, to propose an essential structure of the experience of older adults with asthma or COPD with mHealth. Direct quotations were included to support the analysis [29].

### Strategies to Leverage Qualitative Findings

Following our qualitative analysis, we summarized our findings related to older adults’ use of airway mHealth, and proposed (post hoc) potential strategies to leverage these findings in future mHealth research interested in optimizing older adults’ use of airway mHealth. Strategies were developed using the Expert Recommendations for Implementing Change (ERIC) project list of implementation strategies [30].

### Ethics Approval

This study was reviewed and approved by the Research Ethics Board at St. Michael’s Hospital, Unity Health Toronto (REB# 20-020). Informed consent was obtained prior to enrolling study participants, describing the minimal risk associated with qualitative research participation. Participant data were deidentified and stored on a local secure server. A grocery gift card for CAD $50 (US $37.81) was provided to participants.

## Results

### Overview

We contacted 35 potential participants, and among them, 20 agreed to be interviewed. Among the 15 who did not participate, 5 did not own a smartphone, and the remaining 10 did not return messages. By the 17th interview, we assessed that we had attained data saturation but completed 3 additional interviews (20 total), to determine that no new themes were developed. All participants were home for the interviews, of which 15 (75%) were in video format and 5 by telephone.

The mean age of participants was 79.8 (range 73-91) years. Participants were balanced between sex and airway diagnosis, predominantly used controller inhalers with infrequent reliever use, were university or college educated, and living in urban areas. Most participants owned both a smartphone and tablet device, which they used daily. Participants predominantly used their devices to search the Internet for asthma and COPD information, such as symptoms and treatment options, medication information, and breathing exercises. Some used preinstalled apps to track daily step counts, provide medication reminders, and record personal health information, but they were generally unaware of other downloadable apps specific to asthma, COPD monitoring, or management. Digital devices used included activity trackers (eg, Fitbit watches), digital oximeters, and digital blood pressure monitors. Other than the oximeter, mHealth devices discussed were used for nonrespiratory purposes. See Table 1 for full demographic details.

Two themes characterized the essence of participants’ experiences with mHealth: (1) mHealth was a means of maintaining independence and (2) apprehension was expressed toward a changing technological health care landscape. See Tables S1 and S2 in Multimedia Appendix 1 for a complete list of representative quotes. There were no clear trends based on participant sex or airway diagnosis for either theme.

**Table 1:** Participant characteristics (N=20).

Characteristics		Values
**Age (years)**		
	Mean (SD)	79.8 (4.4)
	Range	73-91
Female, n (%)		10 (50)
**Airway diagnosis, n (%)**		
	Asthma	9 (45)
	COPD^a^	8 (40)
	Asthma and COPD	3 (15)
**Years since airway diagnosis**		
	Mean (SD)	19.9 (11.2)
	Range	7-40
**Rescue inhaler use, n (%)**		
	≤2 times per week	17 (85)
	≤2 times per week	3 (15)
Controller inhaler use		18 (90)
**Education level completed, n (%)**		
	Elementary school	1 (5)
	High school	3 (15)
	College or trade school	5 (25)
	University	11 (55)
**Geographical location, n (%)**		
	Urban	17 (85)
	Rural	3 (15)
**Mobile devices owned, n (%)**		
	Smartphone	20 (100)
	Tablet	16 (80)
	Digital activity tracker	4 (20)
	Digital oximeter	1 (5)
	Digital blood pressure monitor	1 (5)
**Frequency of use of mobile devices, n (%)**		
	Frequently (a few times a day)	18 (90)
	Sometimes (a few times a week)	2 (10)
**mHealth uses, n (%)**		
	Searching the internet for health-related information	15 (75)
	mHealth apps	6 (30)
	mHealth peripheral devices	6 (30)
	No mHealth use	3 (15)

^a^COPD: chronic obstructive pulmonary disease.

### Self-Monitoring With mHealth Was a Means of Maintaining Independence

mHealth was perceived by participants as a tool that could help them maintain their independence. This was reflected in several ways. First, the convenience and accessibility of using mobile devices to search for or track health information was commonly discussed (n=13). Participants appreciated that health information could be searched for immediately when desired, noting: “The phone, it’s just clearly the most convenient tool. I normally have it with me” (ID 6), and “with my iPhone or whatever you call it, if I want to know something instantly...it’s an easy way to find it out” (ID 9). They also emphasized the portability of their mobile devices: “Because you can do it anywhere, anytime, anywhere you want because you just have...it’s in your pocket” (ID 14).

The ability to use mHealth for self-monitoring was also highlighted, providing participants with reassurance about their health status (n=10). Two participants said:

When I wasn’t sure,... it was good to be able to measure [my heart rate] and say, well no, nothing is showing up there.

ID 3

And another participant said:

I find it great to know where I’m at [with my oximeter], and every once in a while I learn something that I wouldn’t have suspected.... If I wake up in the morning and I’m feeling weird, the first thing I want to know is where am I.

ID 15

Self-monitoring with mHealth also helped participants set targets and maintain their physical activity and exercise levels, with 1 participant declaring:

As you get older it becomes a prime exercise activity and having some structure around it and understanding how you’re doing and whether or not you’re progressing, and that sort of thing, is very useful.

ID 6

The information obtained from using mHealth also facilitated more meaningful interactions with participants’ health care providers (n=11). Information obtained using mHealth allowed them to ask more informed questions during medical visits and independently engage in their care. Participants said:

It gives you the sense that you’re participating in your care,... you feel a little bit more independent looking after yourself.

ID 3

And another participant said:

It helps me to explain myself when I have to visit the doctor...and be a little bit more knowledgeable of what I really need to know, or the questions that I need to ask.

ID 5

Using mHealth also helped participants take a more active role in their care and more easily share health information with their doctors; 1 participant detailed:

I note down my symptoms [in my phone] and then when I go to [my doctor] and I’m sitting in front of her I can just go through them and say, this is what’s been going on.

ID 19

Finally, participants principally valued mHealth as a tool to help monitor or manage active respiratory disease that could threaten their independent health status, rather than something to use routinely (n=16). They explained:

If it would affect my life in a negative way, then yeah, I definitely would [track my respiratory symptoms]. I do that with dialysis, which is a serious condition.

ID 9

And another participant said:

There’s enough going on that you worry about or that you can worry about or whatever that I don’t need to have constant update on stuff that isn’t bothering me.

ID 12

### Apprehension Was Expressed Toward a Changing Technological Health Care Landscape

In direct tension with the value of mHealth in maintaining independence was an apprehension expressed toward the way mobile technology could negatively change the provision of health care. This was primarily reflected in a resistance to changing already established health behaviors not involving mobile technology (n=10). For example, participants said:

I avoid apps. Sorry, I sound like a Luddite, I probably am, but what I have been using meets my needs.

ID 18

And another participant said:

I have a very rigid system and I stick to it. No, I wouldn’t [use my smartphone for medication reminders].

ID 20

Many participants also elaborated that their reluctance to use mHealth was related to concerns about having to learn how to use new technology. One participant declared:

I’m not going to twist my brain with this stuff. That would be very confusing to me, and when I don’t understand it, I feel like an old fart who doesn’t understand modern technology. I try to avoid that by not digging deep into it.

ID 9

Participants were also wary of mobile technology replacing in-person visits (n=8). One participant claimed:

Nothing can substitute for human contact, person-to-person contact. Technology is a double-edged sword. It has made things more convenient, but it has also depersonalised therapeutic care.

ID 19

They also felt that more serious medical issues required face-to-face contact instead of technological solutions. Participants stated: “If you’ve got a problem you want to talk to somebody face-to-face” (ID 8) and “If I’m feeling sick, I cannot transfer my symptoms by phone” (ID 9).

Many participants also discussed a lack of confidence using mHealth or raised concerns related to potential fear and anxiety that could result from mHealth use (n=9). For some, this anxiety was related to mHealth data being an unwanted reminder of their medical conditions.

I didn’t look at [my heart rate on the Apple watch], because then I might think that I’m sick. Well, I’m not sick, and I don’t want to think about it.

ID 1

Others were worried that they would feel insecure if they were unable to effectively use mHealth technology.

I think it’s a confidence thing more than anything else [preventing me from trying health apps]. I don’t want to frustrate myself, don’t want to be embarrassed.

ID 15

Interestingly, health data privacy and security issues were not a common concern for participants.

Finally, participants were reluctant to engage with mHealth apps or devices that didn’t originate from a trusted health care source (n=17), stating:

The iPhone, for example, suggests all kinds of apps in there and then you go down and read it and it says, I don’t know. I think anything to do with health, you trust your doctor.

ID 7

And another participant said:

For me, for my respiratory health, it would have to come from [my lung doctor]. I would not buy something...respiratory related without having it recommended to me by my health professionals.

ID 12

## Discussion

### Principal Results

In this study, we explored mHealth-related perceptions and experiences of older adults with asthma and COPD. Overall, their experiences were reflected in a tension between the perception that mHealth could be used to help maintain independence and an apprehension of ways that mHealth might negatively change established health care experiences.

To our knowledge, our study is the first to qualitatively investigate mHealth use exclusively in patients with asthma and COPD who are aged 65 years or older. Use of mHealth was common in our sample of participants who owned a smartphone, with 85% (17/20) of participants using their devices for health-related purposes. This finding is in keeping with an observed trend toward increased use of digital health technology in older adults with chronic diseases [18,20]. However, we found that the extent of mHealth use was limited and that participants generally lacked awareness of downloadable apps and other connected devices specifically designed for asthma and COPD. This result aligns with findings from a recent cross-sectional Canadian survey of older adults in which the authors reported that only 11% of the 682 respondents aged 65 years and older had recently downloaded and used a health app, and only 12.2% owned and used smart devices or wearables [31]. Despite interest and high mobile technology use, the full potential of airway mHealth does not appear to be realized among older adults.

The perception that mHealth can help users maintain independence has been previously reported as a facilitator to health technology use among older adults with chronic conditions [20]. Our study adds to this evidence and provides further contextualization in airway disease, highlighting the importance of convenient and accessible disease self-monitoring and self-knowledge resources to support the autonomy of older adults with asthma and COPD. Notably, the older adults in our study primarily used mHealth for self-monitoring purposes, and did not use mHealth for respiratory disease self-management (eg, for action plans, inhaler reminders, or trigger avoidance), contrasting with qualitative studies of predominantly younger participants with asthma [32] and of telemedicine interventions for COPD, where self-management was considered an important feature of airway health technology [33]. As older adults with asthma and COPD are known to have lower levels of inhaler adherence and self-management skills, such as action plan use and environmental control [14,34], and have shown interest in mHealth self-management tools in other qualitative work [35], this represents an important target for future mHealth solutions that is not currently being addressed in our participant population. Another challenge we identified was that older adults only perceived mHealth to be useful for active respiratory disease states. Patients across ages often misperceive chronic respiratory disease as episodic and intermittent in nature, and this illness perception can disrupt routine self-management behaviors and exacerbation prevention targeted by mHealth [34,36,37]. Potential strategies to leverage these findings are presented in Table 2. These proposed strategies are based on established implementation strategies and provide a blueprint to help future airway mHealth researchers design testable implementation strategies specifically targeting the perspectives and needs of older adults with asthma and COPD identified in our qualitative analysis.

**Table 2:** Potential strategies to increase airway-specific mHealth use in older adults.

Main qualitative findings	Strategies to leverage or overcome findings^a^
Limited awareness of full mHealth options	Outreach from health care practitioners and institutions should inform older adults of validated and safe mHealth options (eg, web-based catalogues, educational materials, recruiting local opinion leaders and champions, academic partnerships)
Use of mHealth principally confined to preinstalled smartphone or tablet apps	Develop mHealth interventions that leverage mobile technology already being used by older adults (eg, spirometry from smartphone microphones [38]) to enhance uptake and adherence
Minimal concern for privacy of health data with mHealth use	Ensuring data security and privacy of mHealth are essential, but focusing on these details may not strongly encourage older adults use (compared to younger adults, among whom privacy concerns are more frequently raised [20,39])
Appreciated convenience and accessibility of mHealth	Convenience, accessibility, and portability of mHealth should be emphasized for older adults through patient outreach, training, and educational materials
Use of mHealth primarily for self-monitoring purposes	Self-monitoring capabilities of mHealth should be emphasized for older adults (adapting implementation to older adults needs or preferences)
No use of mHealth for self-management purposes (ie, action plans, inhaler adherence, environmental controls)	If the patient has a low level of self-management, explore barriers and propose mHealth as a potential solution if appropriate (engaging patients as active participants)
Facilitation of active and informed participation in care with mHealth	Explore desire for more active and informed involvement in care and propose mHealth as a means of patient empowerment
Perception of mHealth as useful only for managing active respiratory disease	Patient education addressing illness perception should be provided, particularly among older adults where health literacy level is lower [14,36]
Resistance to changing established health care routines or habits with mHealth and learning new technology	If possible, introduce mHealth solutions early after disease diagnosis Tailor implementation to older adults by providing older adults with clear information about the expected benefits of changing current habits with mHealth if appropriate [40] Training and educational resources specifically designed to facilitate mHealth use among older adults
Concern that mHealth would replace in-person visits	Focus on mHealth solutions that are designed to enhance and not replace in-person care for older adults [33] (tailoring implementation) Emphasize that in-person care options are always available when using mHealth
Fear and anxiety of constant reminders of poor health with mHealth or of misusing mobile technology	Facilitate structured and ongoing training opportunities for older adults to practice with mHealth interventions and troubleshoot usability barriers [40] Provide clear guidance around appropriate monitoring frequency and how to respond to and interpret mHealth data generated (and how or when to relay data to providers)
Reluctant to engage with mHealth if not recommended from a trusted health care source	Health care practitioners should discuss validated mHealth options with older adults Promotion of objective and transparent criteria for mHealth evaluation (eg, validated checklists) Professional respiratory societies and patient groups should provide guidance around safe and validated mHealth options, with specific outreach targeted to older adults

^a^It is important to recognize that some older adults may not want to incorporate mHealth into their care routines. In these cases, care gaps should be approached in line with patient preferences.

In direct tension with the perceived value of airway mHealth in older adults was their apprehension toward the ways in which mHealth could change their health care experiences. Resistance to change has been previously described as a barrier to health technology use in older adults with chronic diseases, associated with reluctance to adopt new skills and engage with technology [18,20]. This stands in contrast to younger patients with airway disease, who are generally more receptive to integrating new technology into their health care routines [41]. Our study highlights several potential sources of this apprehension, including reluctance to changing established self-monitoring routines; concern that mHealth may be used to reduce or replace in-person care; fear, anxiety, and lack of confidence around mHealth use; and reluctance to accept mHealth that is not endorsed or recommended by trusted health care sources. Other than concerns about mHealth replacing in-person care and anxiety about being required to use mHealth [33,42], these findings are novel compared to qualitative studies of younger adults with airway disease, where apprehensions around mHealth use have focused more around privacy or security and technological reliability [39,41,43]. Strategies to address these apprehensions specific to older adults are proposed in Table 2, as detailed above. However, it is also important to respect patient autonomy, and recognize that some older adults may ultimately not want to incorporate mHealth into their care routines despite potential benefits.

### Limitations

Our study has several limitations. We required smartphone ownership, which likely biased our sample toward a more digitally engaged subset of older adults. This was necessary to answer our research question, as we were interested in the experiences and perspectives of current mHealth use rather than abstract opinions. Smartphone use among older adults is also rising rapidly, with 54% of adults aged 65 years and older owning a smartphone in Canada in 2020, which is a more than 10% increase since 2018 [44]. Our sample was also predominantly urban living and university or college educated, and we did not address important emerging issues related to the cultural and socioeconomic dimension of digital health equity. Finally, our sample was recruited from an existing research database at a single urban tertiary care center. This may have biased our sample toward those more willing to participate in research, but also toward those with more significant respiratory disease, where the potential future impacts of mHealth interventions are particularly valuable.

### Conclusions

By the year 2050, more than 20% of the world’s population will be over 60 years old, nearly double what it is today [45]. With these shifting demographics, a growing number of older adults will be living with asthma and COPD, and mHealth innovations have the potential to help older patients to more effectively manage their chronic respiratory disease. We found that a greater understanding of the perspectives and experiences of older adults with asthma and COPD in their use of mHealth provides valuable insights into how we might further improve and expand its reach. These lessons are applicable immediately and can be evaluated objectively in future airway mHealth research, and then implemented at clinical and health policy levels. We must also keep them in mind as new mHealth innovations emerge to help treat the growing number of older adults with airway disease.

## Appendix

Multimedia Appendix 1 Further details on qualitative methods and patient recruitment, patient interview guide, and representative quotes.

## Abbreviations

COPD: chronic obstructive pulmonary disease
COREQ: Consolidated Criteria for Reporting Qualitative Research
ERIC: Expert Recommendations for Implementing Change
mHealth: mobile health

## Data Availability

The data sets generated during and/or analyzed during this study are available from the corresponding author on reasonable request.
