# Supplementary material for: Exploring the Perspectives and Experiences of Older Adults With Asthma and Chronic Obstructive Pulmonary Disease Toward Mobile Health: Qualitative Study
Source: J Med Internet Res. 2023 Aug 22;25:e45955. doi: 10.2196/45955 (PMC10481221; doi:10.2196/45955)
Supplement: Multimedia Appendix 1 [file jmir_v25i1e45955_app1.docx]

**Considering the influence of an active researcher in phenomenology through reflexivity**

The principle of reflexivity is captured in Giorgi’s interpretation of phenomenological analysis through the “bracketing” of one’s preconceived notions of the phenomenon being studied. The purpose of bracketing is to promote that the researcher remains as presuppositionless as possible and fully open to participant experiences.^26^ In order to accomplish this, AK kept a research journal, documenting his perspective and reflected on this critically throughout data collection and analysis. AK’s previous clinical experience treating older adults with asthma and COPD was also considered. In particular, the assumption that older adults would have less interest in mHealth based on decreased familiarity and experience with mobile technology was critically interrogated throughout the study.

**Further details regarding the patient database used for patient recruitment**

The Respirology Patient Recruitment Database at St. Michael’s Hospital, Unity Health Toronto, is an REB-approved database created to inform prospective research participants about upcoming research they may be interested in and eligible for. Inclusion criteria are a diagnosis of asthma, chronic obstructive pulmonary disease, or bronchiectasis. There are no exclusion criteria. Potential participants are invited to join the database by a Respirologist at St. Michael’s Hospital who follows them clinically. The database includes name, sex, date of birth, hospital record number, respiratory disease, preferred contact information, which physician follows them clinically, and preferences for contact methods/times.

**Patient interview guide**

1. Experience with mHealth technology:
   1. Tell me what kind of smart devices you have currently and what you use them for? (probe about specific devices they may have)
   2. Can you remember the last time you used it for something related to your respiratory health? Or: can you tell me about a time in the last year you used your device for something related to your respiratory health?
   3. Tell me about the reason you used it?
   4. Tell me how you used it, was it alone or with help?
   5. What was it like for you to use your device in this context?
      1. If having difficulty answering: How easy was it to use? How difficult? How useful did you find it? What helped you to use it or hindered your use of it? What did you hope to gain out of using it?
   6. How did your experience make you want to continue using your device in the future?
   7. Tell me about how others around you (your family members or friends) or involved in your health (your healthcare team members) influenced your device use
   8. Tell me about any technical support you needed or problems you ran into when using your device in this way
2. Aging and mHealth questions:
   1. Can you tell me about a time when you used your device for a health-related purpose, and that your age influenced your interaction with it?
      1. If participant having difficulty describing interactions, can ask:
         1. What was it like for you to access health information on your device? Was it easy or hard?
         2. Did you use your own device, a friend’s/family member’s, a hospital/clinic’s? How usable was it? Was there an application that needed to be used?
         3. Can you tell me about a time when using your device improved your health or healthcare? Or when it made it worse?
   2. Do you feel like applications on your device and your device itself are designed to be usable for people of all ages?
   3. Can you think of any ways that having asthma/COPD as an older adult would affect your use of mobile health technologies?
      1. i.e. medication needs, doctor or hospital visits, communication with family and healthcare teams about your health
   4. Do you ever get help using your device for health purposes? Who helps you, family/spouse/caregivers/other?
   5. Are you a caregiver to someone else? Do you use your smart device in your role as a caregiver, and can you tell me about that? What about for spouses/partners?
3. Mobile technology use in general – emphasize that we are now talking about general use, and not only health-specific use:
   1. Take me through a day of using your smartphone or tablet at home
   2. How/when/why (ask each one separately) do you use smartphones or tablets? How long have you been using them? Do you find it easy or hard to use? Tell me about that.
   3. Do you feel like these technologies are usable for people of all ages? If not, can you tell me about a specific time when you felt they weren’t personally?
   4. Do you use these technologies alone or with other people? Tell me about that – address social expectations around use (are you the first one to use it, or do you follow others)
   5. How do you think getting older affects your use of mobile technologies?
      1. Are you more interested or less in using specific devices? Are there specific things you think it is useful for? Do you find it easy or hard to use? And why?
   6. How do you feel when you take your phone out in public?
   7. Are there status issues around it? Do you feel modern, do you have a new phone, does it define you?
   8. What about affordability, does this influence your device use?
   9. What are your overall feelings towards your current device?
   10. Do things you see/read in the news influence your device use or what you think about them? How?
   11. Do you use social media on your mobile device, thoughts about social media in general?
   12. Do you remember when you first started using mobile technology?

**Table S1. Using mHealth to maintain independence quotes**

|  | **Representative quotes** |
| --- | --- |
| Convenience and accessibility | - Used to be that one went into a lab or a physician’s office to get tested regularly, checked regularly and nowadays, a lot of these things you can just measure yourself and then you can forward them. So, that seems to me like it would be a big plus for everybody (ID 3) - It’s easy and availability, I can answer emails or send them at anytime. Flexible timing and convenient (ID 4) - We were able to send pictures and he was able to prescribe and we were able to, back and forth, tell him what was happening when it finally was okay. I found it great communicating by the iPad and taking pictures […] I found it was great. We didn’t have to go in the car, spend that time, park, go in, sit (ID 5) - The phone, it’s just clearly the most convenient tool. I normally have it with me (ID 6) - Yes, we have [used my phone for virtual visits]. At least with that, they’re on time most of the time. When you go to their office you sit three for half an hour or 45 minutes and they can probably solve a lot of problems instantly. I think from that point it’s good and you can always go there if they think it’s worth going down there (ID 7) - With my iPhone or whatever you call it, if I want to know something instantly […] it’s an easy way to find it out (ID 9) - It’s much easier to use my iPad. It’s more of where I go to search things (ID 12) - The portability of the iPad particularly and just being able to carry your phone around with you was nice. And then if I’m going away somewhere or going on the bus, it’s easy to carry the iPad (ID 13) - Because you can do it anywhere, anytime, anywhere you want because you just have … it’s in your pocket. If you have a few minutes, you can look and see what’s going on (ID 14) - I carry them upstairs, downstairs with me, can use them all the time, access and it’s very quick (ID 16) - It’s very helpful to be able to connect that quickly with whoever you need to or find out information quickly (ID 17) - To me your phone is like an appendage. So it’s right there. And the phone has certainly made [tracking symptoms] much, much easier (ID 19) - It’s usually handy and it’s portable and I can do it wherever I am (ID 20) |
| Reassurance provided through self-monitoring | - It’s simply that the device has not got any readout on it, itself, it strictly talks to the app on the iPhone or tablet. It stores data, so you can compare over time. It doesn’t try to interpret the data. It tries to do a little bit more than just produce the two blood pressure readings and the pulse rate, it tries to also spot arrhythmia. […] When I wasn’t sure, […] it was good to be able to measure [my heart rate] and say, well no, nothing is showing up there (ID 3) - It would be interesting to know how much oxygen I’m taking, getting in. Because sometimes in the weather you puff more when you’re out than when you’re in. And depending on the weather, it’s more hard on your lungs than not […] where you could look and go, oh, I’m only getting 80%, oh my god. I’ll sit down (ID 4) - It sets broad targets which you can ignore or not. It has reminder features. It tells you what your average has been over a period of time, that sort of thing which is useful. I’m more just focused on a day-to-day where I have certain targets for activity. I just like to track myself against that […] As you get older it becomes a prime exercise activity and having some structure around it and understanding how you’re doing and whether or not you’re progressing, and that sort of thing, is very useful (ID 6) - Well, I use my Fitbit continuously because I sleep with it, it tells me my sleep and it gives it in points. And it also listed if it's fair sleep or poor sleep or a good sleep or an excellent sleep. And I can feel the difference in my energy level and everything if I get my eight hours, I’m very active physically so I need that (ID 7) - I have a heart condition, and yeah, I keep track of these things, my blood pressure and this kind of thing. I keep these on record here (ID 9) - So my average for the week has only been 794 [steps], and one of the reasons for that is we’ve had company here for a week. But if you look at over the month, and you go back to … there’s some days when I did 4,500 steps. It shows you all that, you can keep an average over months even, six months. There’s lots of information there, and I like information, data, so it’s interesting to look at that (ID 14) - I find it great to know where I’m at [with my oximeter], and every once in a while I learn something that I wouldn’t have suspected […] If I wake up in the morning and I’m feeling weird, the first thing I want to know is where am I (ID 15) - I’m concerned about my health. I know it’s important to do a certain amount of walking and I thought, okay, it’s good to have it tracked. […] When I have a sense of something going on, I can check and sure enough my body sense is not that far off (ID 16) - Because as I degenerate, I may not remember what I did last Tuesday. And if this technology can help track it for me, and then I sit down with you as a person, I sit down on a chair in front of you and show you all of that information, wonderful. It’s an assist and a compensation to my normative degeneration (ID 19) - I use my Fitbit for trying to gain a certain number of steps and then I check my sleep in the morning and look at, for what it’s worth, look at the respiratory pattern that comes out at the bottom of your sleep for the night and different things like that (ID 20) |
| Facilitating participation in care and being an informed patient | - It gives you the sense that you’re participating in your care, […] you feel a little bit more independent looking after yourself (ID 3) - Yeah, it’s like, oh, I’ve heard of that [from online searching], yes, tell me about this. Or I’ve heard about this, would that be any good for me. It’s always extra information (ID 4) - It helps me to explain myself when I have to visit the doctor […] and be a little bit more knowledgeable of what I really need to know, or the questions that I need to ask (ID 5) - I think more and more we’re understanding that … well it sounds obvious, but your health is your own responsibility. Your medical condition and medical health is more and more your own responsibility. And I think there is less and less handholding from health professionals. And so, I think it will just increasingly be offloaded, if you like, so yeah, I think it’s increasingly important [to use personal health technology] (ID 6) - If [my doctor] said oh, I think you should take this puffer instead of that one I might go in and research that a little bit more than what we talked about in our meeting (ID 12) - I’ve created a Word document that lists all my medications that I take and any hospital admissions for the past seven or eight years. So, I’ve got that, and I took a photograph of the medications and that’s on the iPad and it’s on my phone. Because you go to a hospital Emergency Department and the first thing, they ask is what medications are you on (ID 13) - Because one can [look up health information] independently without having to trouble other people for it and you can list your own questions or research them [and] it makes me aware. So, if there’s something that’s off, then I say, okay, you’ve got to pay attention to this. If it’s fine, then I don’t have to pay attention. It helps me to better focus (ID 16) - I note down my symptoms [in my phone] and then when I go to [my doctor] and I’m sitting in front of her I can just go through them and say, this is what’s been going on. (ID 19) |
| Use only for active respiratory disease | - I read of blood sugar monitors and that sounded like something that would be possibly helpful or relevant for me, perhaps for a lot of older people. Nothing to do with breathing, but that’s mainly because I have really a mild case of asthma […] the other conditions, because they make life difficult in some ways, there, I’ve looked up much more, read much more (ID 3) - I really didn’t look up asthma as much as other […] medical issues that I have been having (ID 5) - [I used my step counter app] this past winter because I had some other problems. I had a rib injury and so, I had fairly restrictive breathing, cold weather and mask and all of that. Walking was difficult but understanding why it was difficult, maybe the impact of temperature, humidity, factors like that help diagnose maybe or helped me understand why a particular day was good or difficult […] I should mention something that I did get online, and the phone actually, which is very useful to me, it was whenever I had difficulty over the winter with the rib injury. My breathing was very shallow, so I was getting congestion and I found a set of breathing exercises which turned out to be fairly helpful (ID 6) - I have asthma, but it’s not very severe. I don’t find it very difficult to live with it […] If it would affect my life in a negative way, then yeah, I definitely would [track my respiratory symptoms]. I do that with dialysis, which is a serious condition (ID 9) - I haven’t really seen the need [to track my steps or heart rate], you know, my heart seems to be okay, and my … You know, the things … You know, I suppose if you talked about respirology and I had a serious asthma or something like that, it might be good to be able to track how it was doing or something. I don’t have serious asthma so, yeah (ID 10) - Too much information that I don’t need. Yeah, there’s enough going on that you worry about or that you can worry about or whatever that I don’t need to have constant update on stuff that isn’t bothering me” (ID 12) - I’m using oxygen 24/7 that doesn’t bother me. If it’s absolutely necessary for your health, sure you’re going to do it. You’re going to do it […] To me, it’s not useful [to track my oxygen levels with mHealth]. As long as I’m feeling okay, I’m not going to worry about it (ID 13) - My health problem is breathing, I know the solution for it is I have to monitor my exercise, I have to take the puffer, everything else (ID 14) - I have looked up asthma [on my smartphone]. More recently, I’m thinking I would like to find out if there are any breathing exercises that I can do that would improve my situation in asthma. Not just breathing exercises, are there things I should be aware of that would improve. Therefore, I’m trying to search for sites (ID 16) - Generally speaking, my body is working well, but if it started, and it’s going to start going downhill, of course, anything that would help me delay that, I'll go anywhere to look it up, of course, and obviously, the internet is the best place, it’s the fastest place (ID 18) - I know that cardiology and Apple watches there is something where you can kind of dial in your own cardiogram or rhythm or whatever. Which I find very interesting but fortunately I don’t have major issues in cardiology, so it’s never been applicable (ID 20) |

**Table S2. Apprehension towards mHealth and change subthemes and quotes**

| **Subthemes** | **Representative quotes** |
| --- | --- |
| Resistance to changing established self-monitoring and management habits | - If you were somebody that liked trying new things, it doesn’t matter how old you are. But if you weren’t and you had a very predictable, safe life, which is perfectly fine for some people. They don’t want anything new to disrupt the harmony of their current lives (ID 4) - I guess it’s the comfort level. I’m still of the old school. I’m still comfortable writing rather than [using the smartphone]… but I’m trying, trying to change over, bit by bit (ID 5) - But you’re reluctant, I’m not going to pay more for the iPhone than I paid for this one, even though it’s better, because you’re going to have to learn again (ID 7) - I don’t know. I never look [online health resources] on my cell phone. If I had an iPad maybe, I might do that. I’m sort of set in my ways (ID 8) - I keep it simple for myself. I’m not very curious about new things that come onto the market. I’m not going to twist my brain with this stuff. That would be very confusing to me, and when I don’t understand it, I feel like an old fart who doesn’t understand modern technology. I try to avoid that by not digging deep into it (ID 9) - Because my history is I’ve never been able to master [technology], therefore I won’t be able to master it, so that maybe I shouldn’t try (ID 11) - I have a file that is my health and I just write it and that’s about all that’s in there […] At least with a piece of paper I know it’s there and I can look back and say oh, I forgot that I had a cold that year and that my temperature and this and that and I took my emergency puffer. But if I had a place to store it, like I have notes and stuff like that on my iPad, but I don’t write anything in there (ID 12) - I’m fortunate that Word documents that I use to track my daily med events and so on. Because of my age and my background, as long as those things are working, that’s the simplest way for me to deal with it […] for me this is so simple that it’s actually easier than dealing with technology (ID 15) - I avoid apps. Sorry, I sound like a Luddite, I probably am, but what I have been using meets my needs (ID 18) - I have a very rigid system and I stick to it. No, I wouldn’t [use my smartphone for medication reminders] (ID 20) |
| Concern that mHealth may replace in-person care | - Depending what it’s about. It could well work with a combination, you know, he wants to talk to you about something you could do it on a zoom. And then, or he got some results back and he wants to.. But if you got a problem.. you want an in person visit (ID 2) - If you’ve got a problem you want to talk to somebody face-to-face (ID 8) - If I’m feeling sick, I cannot transfer my symptoms by phone (ID 9) - I kind of like to go in. I like people face-to-face. I will be so happy when I'm face-to-face again (ID 10) - I would probably just ask for an appointment with [my doctor], and talk about it more in detail (ID 11) - I think that the idea of going to virtual … okay, so [my doctor] phones and says, how are you doing? I’m fine. Okay see you next year. If that’s going to happen I would prefer an in person appointment and to have a breathing test and all that (ID 12) - I think there are some times when [technology that replaces in-person visits] does interfere with the sort of practical aspect of medicine, that people take it into their own hands, to be able to do diagnoses and then even go as far as trying to do some treatments, that may or may not be effective or even harmful. So, I think there can be some real downsides to empowering people with technology to become experts in medicine (ID 17) - I’ll go back to my old adage, nothing can substitute for human contact, person-to-person contact. Technology is a double-edged sword. It has made things more convenient, but it has also depersonalised therapeutic care (ID 19) |
| Lack of confidence, fear, and anxiety related to mHealth and technology use | - I didn’t look at [my heart rate on the Apple watch], because then I might think that I’m sick. Well, I’m not sick, and I don’t want to think about it (ID 1) - I’m not 100% comfortable […] I feel inferior, like I just don’t get it as fast as they do. Possibly, because I have been able to lean on my husband, I haven’t advanced as well as I should have in the technology of using the iPad (ID 5) - There's anxiety in when I lose it, like, the thing goes away, I press the wrong button, I whatever. It’s not like watching one of my sons who goes bleh bleh bleh and then it’s done again, you know, there it is again. I'm like, ah, now I've killed it and I can't get back. Yeah, so there's some anxiety with this stuff (ID 10) - I guess that fear of failure is so strong in me about mechanical things, that I would probably get really depressed or angry or both, and say, I can’t do this, that’s all there is to it (ID 11) - I think it’s a confidence thing more than anything else [preventing me from trying health apps]. I don’t want to frustrate myself, don’t want to be embarrassed (ID 15) - I think [self-monitoring with mHealth] might be more than I’d want to necessarily know or have to deal with all the time. You’re right, I think that would increase anxiety more than anything else, which is not going to be helpful at all (ID 17) - In my case, I probably started out with that sense of lacking in confidence, being a bit awed by [mobile technology] (ID 19) - I’m not sure that I would [use mHealth] on my own because I’ve got a number of different things that are on the go medically. I’m at the point where I’m taking numerous medications. And I’m a little bit leery of adding or subtracting because the scale is very finely tuned. It’s balanced at this point and I’m not a risk taker as far as that goes and I’m just not anxious to do anything on my own particularly that I can think of (ID 20) |
| Unwillingness to use mHealth unless it comes from a trusted healthcare source | - Well, if I went to the doctor and they told me that I should eat this, or do that, or try this, or try that, that would work for me. But I don’t really want to go on the internet. Because when you go on the internet, how do you know that it’s right? I’ve read some stuff and it’s absolutely appalling (ID 1) - If physicians would routinely tell patients, look, this issue we’ve been discussing, you can read up more about it if you’re interested and here’s were I suggest you look, these are good sources. That’s clearly not part of current medical education or practice, but I find it easy to imagine how that could be done in a way that was fruitful (ID 3) - I wouldn’t go to the App Store […] and especially if [my doctor] said, or if I got word from [the hospital] saying, with your particular issue this may help you, would you like to check it out? If it came from a bona fide source that I trusted, yes, it would be of value (ID 4) - The iPhone, for example, suggests all kinds of apps in there and then you go down and read it and it says, I don’t know. I think anything to do with health, you trust your doctor (ID 7) - For me, for my respiratory health, it would have to come from [my lung doctor]. I would not buy something […] respiratory related without having it recommended to me by my health professionals.” (ID 12) - If a doctor said to me there’s some apps that would be very beneficial to you, I’d be the first to jump on board, for sure. But that hasn’t happened so far in my experience […] I think I would definitely want to have a recommendation, as opposed to just sort of discovering an online solution to something that is not necessarily been well researched or whatever (ID 17) - I would like [mHealth] to come from a reliable, bona fide source. And once I’m in place with it, then I might develop a sense of autonomy that I can trust this, and I can do that (ID 19) - I mean a recommendation always helps and the kind of trustworthiness of the app and confidentiality and all that kind of stuff. And if it’s recommended by a professional then yeah, I would use it. I’m not sure that I would do anything on my own (ID 20) |
